# Tributyrin Mitigates Ethanol-Induced Lysine Acetylation of Histone-H3 and p65-NFκB Downregulating CCL2 Expression and Consequent Liver Inflammation and Injury

**DOI:** 10.3390/nu15204397

**Published:** 2023-10-17

**Authors:** Smita S. Ghare, Benjamin T. Charpentier, Dushan T. Ghooray, Jingwen Zhang, Manicka V. Vadhanam, Sreelatha Reddy, Swati Joshi-Barve, Craig J. McClain, Shirish S. Barve

**Affiliations:** 1 Department of Medicine, University of Louisville, Louisville, KY 40202, USA; 2 UofL Alcohol Center, University of Louisville, Louisville, KY 40202, USA; 3 UofL Hepatobiology COBRE, University of Louisville, Louisville, KY 40202, USA; 4 Department of Anatomical Science and Neurobiology, University of Louisville, Louisville, KY 40202, USA; 5 Robley Rex VA Medical Center, University of Louisville, Louisville, KY 40202, USA

**Keywords:** alcohol, histone H3 acetylation, p300, SIRT1, CCL2 promoter, tributyrin

## Abstract

Purpose: Chemokine-driven leukocyte infiltration and sustained inflammation contribute to alcohol-associated liver disease (ALD). Elevated hepatic CCL2 expression, seen in ALD, is associated with disease severity. However, mechanisms of CCL2 regulation are not completely elucidated. Post-translational modifications (PTMs) of proteins, particularly acetylation, modulate gene expression. This study examined the acetylation changes of promoter-associated histone-H3 and key transcription factor-NFκB in regulating hepatic CCL2 expression and subsequent inflammation and injury. Further, the effect of therapeutic modulation of the acetylation state by tributyrin (TB), a butyrate prodrug, was assessed. Methods: Hepatic CCL2 expression was assessed in mice fed control (PF) or an ethanol-containing Lieber–DeCarli (5% *v*/*v*, EF) diet for 7 weeks with or without oral administration of tributyrin (TB, 2 g/kg, 5 days/week). A chromatin immunoprecipitation (ChIP) assay evaluated promoter-associated modifications. Nuclear association between SIRT1, p300, and NFκB-p65 and acetylation changes of p65 were determined using immunoprecipitation and Western blot analyses. A Student’s *t*-test and one-way ANOVA determined the significance. Results: Ethanol significantly increased promoter-associated histone-H3-lysine-9 acetylation (H3K9Ac), reflecting a transcriptionally permissive state with a resultant increase in hepatic CCL2 mRNA and protein expression. Moreover, increased lysine-310-acetylation of nuclear RelA/p65 decreased its association with SIRT1, a class III HDAC, but concomitantly increased with p300, a histone acetyltransferase. This further led to enhanced recruitment of NF-κB/p65 and RNA polymerase-II to the CCL2 promoter. Oral TB administration prevented ethanol-associated acetylation changes, thus downregulating CCL2 expression, hepatic neutrophil infiltration, and inflammation/ injury. Conclusion: The modulation of a protein acetylation state via ethanol or TB mechanistically regulates hepatic CCL2 upregulation in ALD.

## 1. Introduction

Sustained hepatic inflammation is an important factor in alcohol-associated liver disease (ALD) development and progression. Neutrophil infiltration and monocyte recruitment in the liver are associated with chronic alcohol-induced liver inflammation and injury [1,2]. Chemokines act as chemo-attractants and activators, allowing the recruitment of these neutrophils and monocytes to the liver and inducing an innate and adaptive immune response [3,4]. Among four different chemokine families, the C-C subfamily is the largest, and chemokines such as C-C motif chemokine ligand 2 (CCL2), also referred to as monocyte chemotactic peptide-1 (MCP-1), are reported to be present in high levels in patients with alcohol-associated hepatitis [5,6]. Both plasma levels and hepatic expression of CCL2 have been reported to correlate with disease severity [5]. Importantly, CCL2 knockout mice were protected against alcoholic liver injury by the induction of genes responsible for fatty acid oxidation and inhibition of pro-inflammatory cytokines [7]. Although these studies emphasize the key role of CCL2 in inducing liver pathologies, including hepatic inflammation, the molecular mechanisms that regulate their expression in ALD, particularly in response to alcohol, are yet to be completely elucidated.

Emerging studies have documented the role of the gut–liver axis in ALD in the context of alcohol-induced alterations in gut microbiota, barrier dysfunction, and endotoxemia leading to sustained hepatic inflammation. Alcohol consumption is known to affect short-chain fatty acids (SCFAs) levels, particularly butyrate [8]. Butyrate has been demonstrated to have an epigenetic regulatory role by acting as an HDAC inhibitor [9,10,11]. Additionally, butyrate has been shown to inhibit inflammatory responses [12,13,14]. Tributyrin is a butyrate prodrug consisting of three butyrate molecules that can be released in the circulation upon digestion by gastric and pancreatic amylases and lipases. Experimental studies have shown that tributyrin supplementation exhibits beneficial effects via maintaining gut bacterial diversity and barrier integrity in ethanol-fed animals [15]. Moreover, our previous work showed that oral administration of tributyrin can protect against ethanol-induced alterations in hepatic HDACs expression/activity and promoter histone modifications and can regulate the expression of genes involved in lipid metabolism [16]. Since there is no specific FDA-approved treatment for ALD and novel therapies are urgently needed, the potential role of tributyrin administration as a dietary therapeutic option against sustained hepatic inflammation needs to be investigated.

Epigenetic regulation is critical for gene expression. In particular, a post-translational acetylation modification is known to dictate the transcriptional activation of a given gene, as this modification regulates functions of both histones and non-histone proteins, like transcription factors [17]. Histone deacetylases (HDACs) and histone acetyltransferases (HATs) maintain the acetylation states of these proteins and play a key role in gene transcription [18,19]. We have shown that alcohol alters hepatic HDAC expression in a binge alcohol model [20]. Alcohol is also known to increase the global histone acetylation levels in the liver [21,22,23,24]; however, limited information is available for gene-specific effects.

In the present study, we demonstrated that ethanol-induced pathogenic alterations in acetylation states of promoter-associated histone H3 and essential transcription factor NFκB contribute to the upregulation of hepatic CCL2 expression. Further, tributyrin, a dietary butyrate prodrug, prevents ethanol-mediated modulation of SIRT-1-NFκB-p300 interactions and resultant acetylation changes. 

## 2. Materials and Methods

Animal Model: Eight-week-old male C57BL/6N mice were obtained from Harlan (Indianapolis, IN, USA). All mice were housed in a pathogen-free, temperature-controlled animal facility accredited by the Association for Assessment and Accreditation of Laboratory Animal Care with 12 h light/12 h dark cycles. All experiments were carried out according to the criteria outlined in the Guide for Care and Use of Laboratory Animals and with the approval from the University of Louisville Animal Care and Use Committee (IACUC# 20754). Mice were fed a modified Lieber–DeCarli liquid diet enriched in unsaturated fat (corn oil), which provided 40% of energy from fat, 43% from carbohydrate, and 17% from protein (Bio-Serv Inc., Frenchtown, NJ, USA). Mice were pair-fed a Lieber–DeCarli liquid diet containing either ethanol (EF) or isocaloric maltose dextrin (PF) for 7 weeks. Ethanol was gradually increased for one week, and then, mice were fed the ethanol diet (5% (*v*/*v*)) ad libitum for 7 weeks (EF). The control-pair-fed (PF) mice were given the isocaloric maltose-dextrin-containing liquid diet. For tributyrin treatment groups, both pair-fed (TB) and ethanol-fed (EF + TB) mice received tributyrin (Sigma Aldrich, St. Louis, MO, USA) by oral gavage (2 g/kg, 5 days per week) for 7 weeks as a preventive strategy (EF + TB (7 + 7)) or for the last 3 weeks as an interventional strategy (EF + TB (7 + 3)). The tributyrin-alone group (TB) received oral TB administration (2 g/kg, 5 days per week) for 7 weeks.

Liver Histopathological Examination: For histological analysis, liver sections were fixed in 10% buffered formalin for 24 h and embedded in paraffin. Tissue sections were stained with hematoxylin-eosin (H&E), F4/80+, and CAE staining and examined under light microscopy with 20× magnification. Assays were performed according to the manufacturer’s protocols. 

Assessment of Liver Injury: The liver injury was assessed by measuring alanine aminotransferase (ALT) and aspartate aminotransferase (AST) using commercially available kits and manufacturer’s instructions (Thermo Electron, Melbourne, Australia).

Blood Endotoxin Assay: Serum endotoxin levels were measured using a Limulus Amebocyte Lysate kit (Lonza, Walkerville, MD, USA) according to the manufacturer’s instructions.

MPO Assay: For hepatic myeloperoxidase activity (MPO), liver samples were homogenized (10 μg/200 μL) in 200 mM NaCl, 5 mM EDTA, 10 mM tris, and 10% glycerin and centrifuged at 1500× *g* for 15 min. The MPO levels were measured according to the manufacturer’s instructions (Hycult Biotech, Plymouth Meeting, PA, USA) at 450 nm using a plate reader. Results are expressed as mg of MPO levels/gram of protein, as determined via a Lowry protein assay (Bio-Rad, Hercules, CA, USA)

Hepatic CCL2 Protein Measurements: Liver lysates were homogenized in a lysis buffer (20 mM Tris, 0.25 M sucrose, 2 mM EDTA, 10 mM EGTA, and 1% Triton X-100), and analysis was performed using a U-Plex kit from Mesoscale Discovery (MSD) (Rockville, ML, USA). Over 200 μg of protein was loaded into each well. The plate was read using a MESO QuickPlex SQ 120 imager and analyzed using Discovery Workbench v4.0 software. The assay was performed according to the manufacturer’s instructions.

RNA Isolation and Real-Time PCR Analysis: Total RNA was isolated using TRIzol (Invitrogen, Carlsbad, CA, USA), and cDNA was made using Quanta qScript (Quanta BioSciences, Gaithersburg, MD, USA). The real-time PCR was performed with Quanta Perfecta SYBR green fast mix and an ABI Prism 7500 sequence detection system (Applied Biosystems, Foster City, CA, USA). The relative gene expression was analyzed using the 2^−ΔΔCt^ method by normalizing with TBP (TATA-binding protein) gene expression in all the experiments and is presented as a fold change over untreated/pair-fed, which was set at 1.

Mouse CCL2 mRNA primers

Forward: GGCTCAGCCAGATGCAGT

Reverse: TGAGCTTGGTGACAAAAACTACAG

Mouse TBP mRNA primers

Forward: CCTAAAGACCATTGCACTTCGT

Reverse: GCTCCTGTGCACACCATTTT

Chromatin Immunoprecipitation (ChIP) and qChIP PCR Analysis: The ChIP assay was conducted using the ChIP assay protocol established in the lab [25]. ChIP antibodies directed against anti-acetyl H3K9 (17-615), anti-phospho H3S10 (17-685), anti-p300 (05-257), anti-p65 (17-10060), and anti-RNA polymerase II (17-672) were purchased from EMD Millipore (Burlington, MA, USA). ChIP-PCR primers designed for the regions of the CCL2 were used, and their sequences are detailed below. Data were analyzed as a differential occupancy fold change. ChIP-qPCR results were calculated using the ΔΔCt method, where each ChIP DNA fraction’s Ct value was normalized to the input DNA fraction. The specificity of each ChIP was established using the corresponding isotype-specific control antibodies (IgG).

Three ChIP-PCR primers were designed for each region of the mouse-CCL2 promoter. The primer sequences are as follows:
(Region I) mmCcl2_F1CAAGCACCCTGCCTGACT(Region I) mmCcl2_R1CTCCCGTCTGGCTCTCTG(Region II) mmCcl2_F2TCCCAGGAGTGGCTAGAAAA(Region II) mmCcl2_R2TCCGCTGAGTAAGTGCAGAG(Region III) mmCcl2_F3CATCTGGAGCTCACATTCCA(Region III) mmCcl2_R3GGCAGGTCAGAGGCAGAGTA

Immunoprecipitation (IP) and Western Blot (WB) Analysis: Total nuclear extracts were prepared by lysing liver tissue in a RIPA lysis buffer (25 mM Tris/HCl pH 7.5150 mM NaCl, 1% NP-40, 0.1% SDS, 1 mM DTT, 10% glycerol, 1× protease inhibitor cocktail, 0.5% sodium deoxycholate, 1 mM Na_2_VO_3_, and 10 mM NaF). All IP and WB analyses were performed as described earlier [26]. Detection of Lamine-B1 served as a loading control. Quantification was performed with ImageLab analysis software v6.0.1 (Bio-Rad). The data shown are representative of three separate experiments showing similar results. Primary antibodies for Acetyl-NF-κB p65 (Lys310) (#3045), SIRT1 (#2310), p300 (#86377), NF-κB p65 (L8F6, mouse mAb #6956), NF-κB p65 (D14E12, rabbit mAb #8242), GAPDH (D16H11, #5174), Lamin B1 (D9V6H, #13435), and secondary antibodies anti-rabbit IgG, HRP-linked antibody (#7074), and anti-mouse IgG, HRP-linked antibody (#7076) were purchased from Cell Signaling Technology, Inc. (Danvers, MA, USA).

Statistical Analysis: Data are presented as mean ± SEM for the indicated number of independently performed experiments or 4–6 mice per group. A Student’s *t*-test and a one-way ANOVA with Bonferroni multiple comparison tests were used to determine statistical significance. A *p* < 0.05 was considered statistically significant.

All authors had access to all data and reviewed and approved the final manuscript.

## 3. Results

### 3.1. Oral Administration of Tributyrin Attenuates Alcohol-Induced, Neutrophil Infiltration, Kupffer Cell Activation, Systemic Endotoxemia, and Hepatic Injury

**Figure 1:**
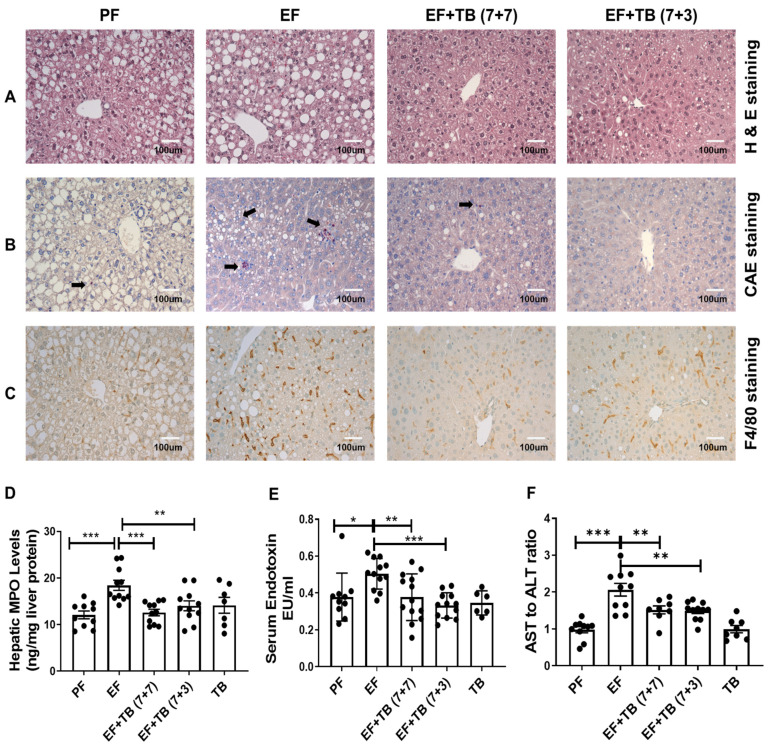
Effect of tributyrin administration on hepatic steatosis, inflammation, and injury in chronic ethanol-fed mice: Mice were fed either a control diet (Pair-fed, PF) or ethanol (ethanol-fed; EF) (5% *v*/*v*)-containing diet. Tributyrin (TB; 2 g/kg) was orally administered to ethanol-fed mice as a preventive (EF + TB (7 + 7)) or interventional (EF + TB (7 + 3)) treatment strategy. Immunohistochemical analysis was performed as (**A**) H&E for steatosis, (**B**) CAE staining for neutrophil infiltration and is indicated by black arrows, and (**C**) F4/80 staining for Kupffer cell activation in mice liver tissue sections frozen in OCT. All images were acquired using a 20× objective and scale bar showing 100 µm. (**D**) Hepatic MPO levels, (**E**) serum endotoxin levels, and the (**F**) AST to ALT ratio were biochemically assessed. Statistical analysis: mean ± SEM. * *p* < 0.05, ** *p* < 0.01, and *** *p* < 0.001 compared with PF or EF using ANOVA with Bonferroni’s test (n = 8–10).

Both preventive and interventional treatment strategies were employed to test the effect of oral administration of tributyrin on alcohol-induced hepatic neutrophil infiltration and Kupffer cell activation in experimental ALD. Initially, a histological assessment was performed to examine the effect of tributyrin administration on markers of hepatic inflammation and injury. In accordance with earlier reports, livers from mice fed ethanol for 7 weeks (EF) had significantly higher hepatic steatosis, as shown by H&E staining (Figure 1A) and neutrophil infiltration, as indicated by CAE staining (Figure 1B). Importantly, tributyrin administration, by both preventive and interventional strategies, inhibited ethanol-induced hepatic steatosis (Figure 1A) and neutrophil infiltration of liver tissue (Figure 1B). Moreover, there was a significant decrease in the hepatic levels of myeloperoxidase (MPO), an enzyme expressed predominantly in neutrophils (Figure 1C). Additionally, as compared to livers from ethanol-fed mice, there was a significant reduction in the number of activated macrophages as seen by markedly fewer F4/80 positive (brown color-stained cells) Kupffer cells in mice administered TB (Figure 1D) with the concomitant decrease in systemic endotoxin levels (Figure 1E) and markers of liver injury (AST/ALT ratio) (Figure 1F).

### 3.2. Tributyrin Mitigates Ethanol-Inducible Increase in Hepatic CCL2 Chemokine Expression

**Figure 2:**
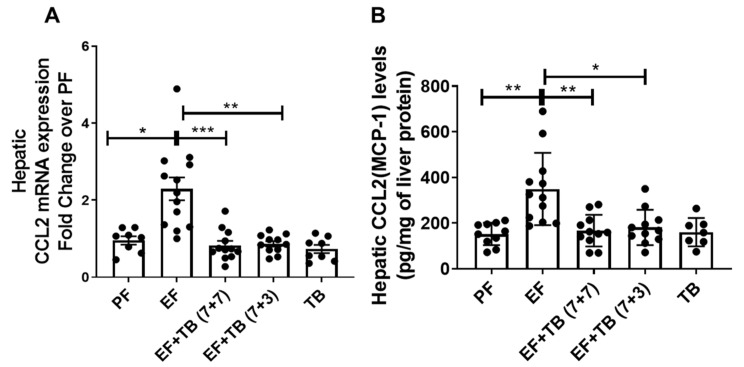
Effect of tributyrin administration on hepatic CCL2 chemokine gene expression in chronic ethanol-fed mice: Mice were fed either a control diet (pair-fed, PF) or ethanol (ethanol-fed; EF) (5% *v/v*)-containing diet. Tributyrin (TB; 2 g/kg) was orally administered to ethanol-fed mice as a preventive (EF + TB (7 + 7)) or interventional (EF + TB (7 + 3)) treatment strategy. (**A**) Hepatic mRNA analysis for CCL2 and TBP (TATA-binding protein) genes was performed using real-time PCR. CCL2 mRNA expression was normalized with TBP and presented as a fold change over PF. (**B**) Liver chemokine CCL2 (MCP-1) protein levels were analyzed using an MSD U-plex assay. The data are presented as a bar graph after normalizing CCL2 levels with total protein concentration. Statistical analysis: mean ± SEM. * *p* < 0.05, ** *p* < 0.01, and *** *p* < 0.001 compared with PF or EF using ANOVA with Bonferroni’s test (n = 8–10).

Chemokines act as a critical mediator for the recruitment of neutrophils and macrophages, and elevated expression of hepatic CCL2 has been well documented to correlate with PMN infiltration and disease severity in ALD [5]. Hence, we next examined the effect of ethanol feeding and tributyrin administration on hepatic CCL2 expression. Our data showed that chronic ethanol feeding caused an increase in hepatic expression of CCL2 at both mRNA (Figure 2A) and protein levels (Figure 2B). Importantly, the increase in the hepatic CCL2 expression in response to ethanol was significantly prevented by tributyrin administration under both preventive and interventional strategies (Figure 2).

### 3.3. Tributyrin Prevents Ethanol-Induced Transcriptionally Permissive CCL2 Promoter-Associated Histone Modifications in the Liver

**Figure 3:**
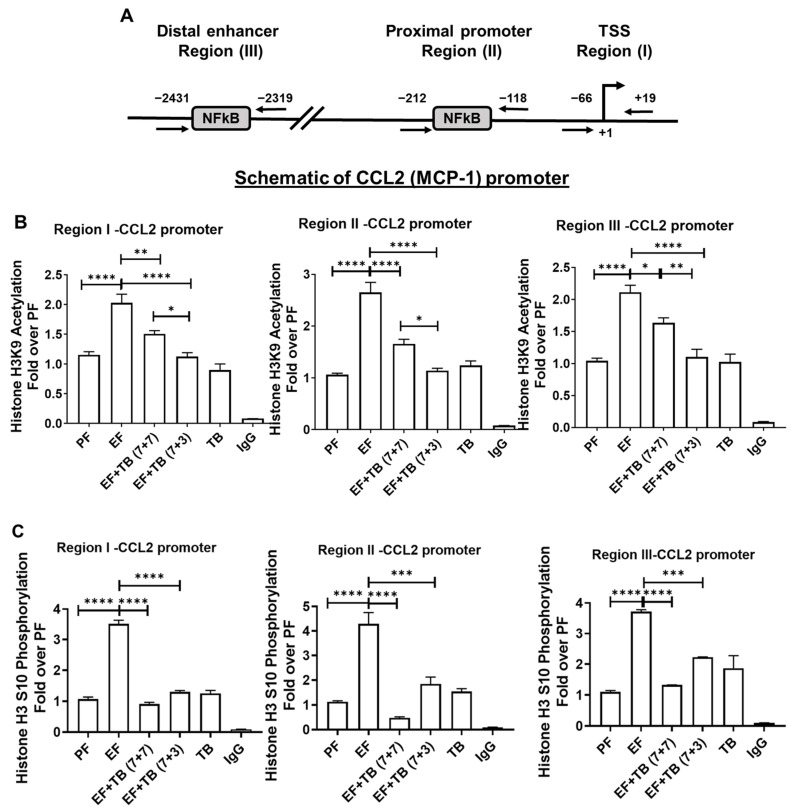
Effect of tributyrin administration on chronic ethanol-induced promoter-associated histone H3 modifications at CCL2 promoter in the liver: Mice were fed either a control diet (pair-fed, PF) or ethanol (ethanol-fed; EF) (5% *v*/*v*)-containing diet. Tributyrin (TB; 2 g/kg) was orally administered to ethanol-fed mice as a preventive (EF + TB (7 + 7)) or interventional (EF + TB (7 + 3)) treatment strategy. (**A**) Schematic of CCL2 promoter: Locations of transcription factor NFκB binding sites and ChIP-PCR primer pairs for analysis of epigenetic modifications are denoted as regions I–III. The coordinate locations shown are with respect to the transcription start site in REFSEQ NM_011333. Hepatic CCL2 promoter-associated histone modifications were assessed by analyzing chromatin that was immunoprecipitated with (**B**) acetylated anti–histone H3 lysine9 (H3K9) and (**C**) phosphorylated anti-histone H3 serine 10 (H3S10) antibodies. Levels of histone modifications were measured using primers specific for regions I, II, and III, as shown in the schematic. Differences are expressed as fold-over PF after normalizing for input DNA. Statistical analysis: mean ± SEM. * *p* < 0.05, ** *p* < 0.01, *** *p* < 0.001, and **** *p* < 0.0001 compared with PF or EF via ANOVA with Bonferroni’s test (n = 5–8).

Epigenetic modifications are known to orchestrate the interplay between the chromatin state and gene expression. To understand whether the post-translational histone modifications occurring at the CCL2 promoter contribute to its gene expression in response to alcohol and tributyrin, a Chromatin immunoprecipitation (ChIP) assay was performed. Promoter-associated histone modifications were investigated via ChIP qPCR at three transcriptionally relevant sites known for NFκB occupancy on the CCL2 promoter [27,28,29]. Region-I interrogated the TSS site (−66 to +19), and Region-II (−118 to −212) and Region-III (−2319 to −2431) investigated proximal and distal enhancer regions known for their NFκB binding on the CCL2 gene promoter (Figure 3A). The specificity of each ChIP was established using corresponding isotype-specific control antibodies (IgG)

The status of histone H3 lysine 9 acetylation (H3K9Ac), which plays a key role in the transcriptional activation of gene expression, was examined. ChIP analysis demonstrated that correspondent to CCL2 gene expression, ethanol feeding significantly increased transcriptionally permissive histone H3 lysine 9 acetylation (H3K9Ac) at all three regions of the CCL2 promoter in murine livers (Figure 3B). Additionally, the effect of ethanol was also examined on another transcriptionally permissive modification. Histone H3 serine 10 phosphorylation (H3S10P), which is also known to be linked with histone H3K9 acetylation was also examined (Figure 3C). The data showed that similar to H3K9 acetylation, ethanol feeding also increased H3S10 phosphorylation at all regions of the CCL2 promoter. In contrast, both preventive and interventional treatment strategies of tributyrin administration significantly inhibited ethanol-induced H3K9 acetylation and H3S10 phosphorylation at all regions of the CCL2 promoter (Figure 3B,C).

### 3.4. Tributyrin Impedes Ethanol-Responsive p300 Recruitment to the CCL2 Gene Promoter

**Figure 4:**
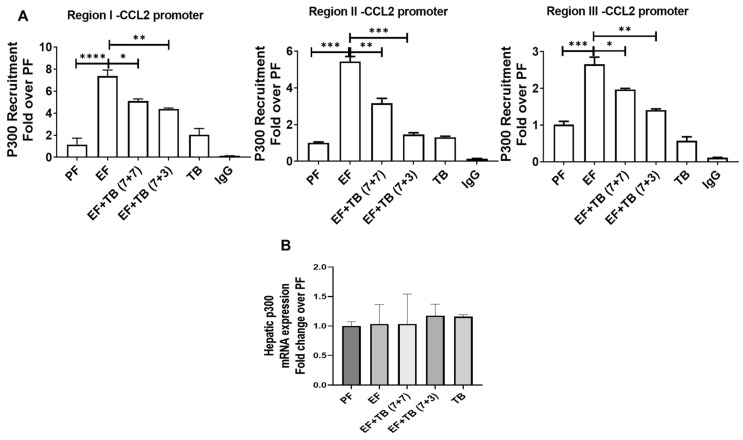
Effect of tributyrin administration on chronic ethanol-induced recruitment of p300-HAT at the CCL2 promoter in the liver: Mice were fed either a control diet (pair-fed, PF) or ethanol (ethanol-fed; EF) (5% *v*/*v*)-containing diet. Tributyrin (TB; 2 g/kg) was orally administered to ethanol-fed mice as a preventive (EF + TB (7 + 7)) or interventional (EF + TB (7 + 3)) treatment strategies. ChIP-qPCR quantification from (**A**) anti-p300 immunoprecipitated chromatin was performed. Differences are expressed as fold-over PF after normalizing for input DNA. (**B**) Hepatic mRNA analysis for p300 gene expression was performed using real-time PCR after normalizing with the TBP gene and expressed as a fold change over PF. Statistical analysis: mean ± SEM. * *p* < 0.05, ** *p* < 0.01, *** *p* < 0.001, and **** *p* < 0.0001 compared with PF or EF via ANOVA with Bonferroni’s test (n = 5–8).

Histone acetylation is regulated by histone acetyltransferases (HATs), and p300 is a known HAT that has been previously reported to increase histone acetylation at the CCL2 promoter [30,31]. Hence, we examined the changes in the p300 binding in response to ethanol and TB treatment. Similar to changes in histone H3K9 acetylation changes, ethanol feeding increased the p300 binding by ~7-fold at Region I, ~5-fold at Region II, and ~2.7-fold at Region III (Figure 4A) over PF. Interestingly, although being known as an HDAC activity inhibitor, tributyrin administration reduced p300 binding under both treatment strategies; however, the effect was more significant for interventional TB treatment (Figure 4A). There was no change in hepatic mRNA expression of p300 for any experimental groups. (Figure 4B).

### 3.5. Tributyrin Attenuates Ethanol-Induced Enhanced Binding of Transcription Factor NFκB at the CCL2 Promoter

**Figure 5:**
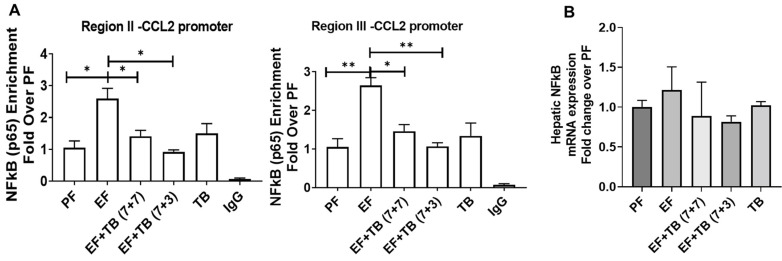
Effect of tributyrin administration on chronic ethanol-induced recruitment of the transcription factor NFκB at CCL2 promoter in the liver: Mice were fed either a control diet (pair-fed, PF) or ethanol (ethanol-fed; EF) (5% *v/v*)-containing diet. Tributyrin (TB; 2 g/kg) was orally administered to ethanol-fed mice as a preventive (EF + TB (7 + 7)) or interventional (EF + TB (7 + 3)) treatment strategy. (**A**) ChIP-qPCR quantification from immunoprecipitated chromatin using anti-NF-kB subunit RelA/p65 antibody was performed. Differences are expressed as a fold-over PF after normalizing for input DNA. (**B**) Hepatic mRNA analysis for NFκB was performed using real-time PCR after normalizing with the TBP gene and expressed as a fold change over PF. Statistical analysis: mean ± SEM. * *p* < 0.05, and ** *p* < 0.01 compared with PF or EF via ANOVA with Bonferroni’s test (n = 5–8).

NFκB is one of the major transcription factors for CCL2 expression. Additionally, it is known that NFκB interacts with the co-activator protein p300 as part of the transcription initiation complex and recruits the complex to the target gene promoters [32]. Hence, we next examined the recruitment of NFκB to the CCL2 promoter via ChIP assay. Commensurate with increased p300 binding and upregulation of CCL2 mRNA expression, ethanol-exposed livers showed enhanced recruitment of NFκB at both proximal and distal enhancer regions of the CCL2 promoter. Moreover, TB treatment prevented NFκB binding to both proximal and distal enhancer regions of the CCL2 promoter (Figure 5A). There was no change in the mRNA expression of NFκB under these experimental conditions (Figure 5B).

### 3.6. Tributyrin Modulates the SIRT1-NFκB-p300 Interaction and Transcriptional Activation of NFκB via Decreasing K310-Acetylation of the Nuclear RelA/p65 Subunit of NFκB in Ethanol-Fed Livers

**Figure 6:**
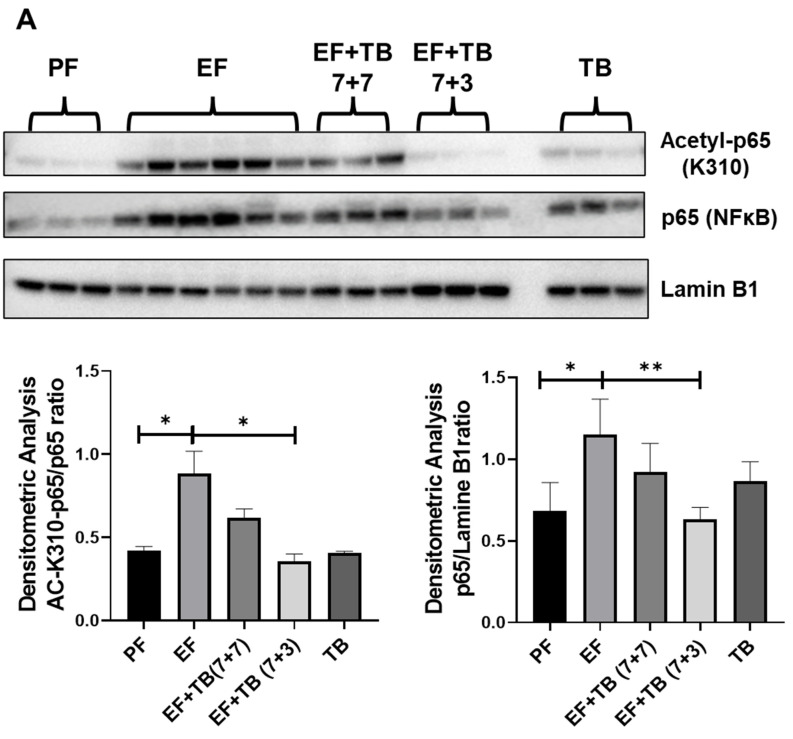

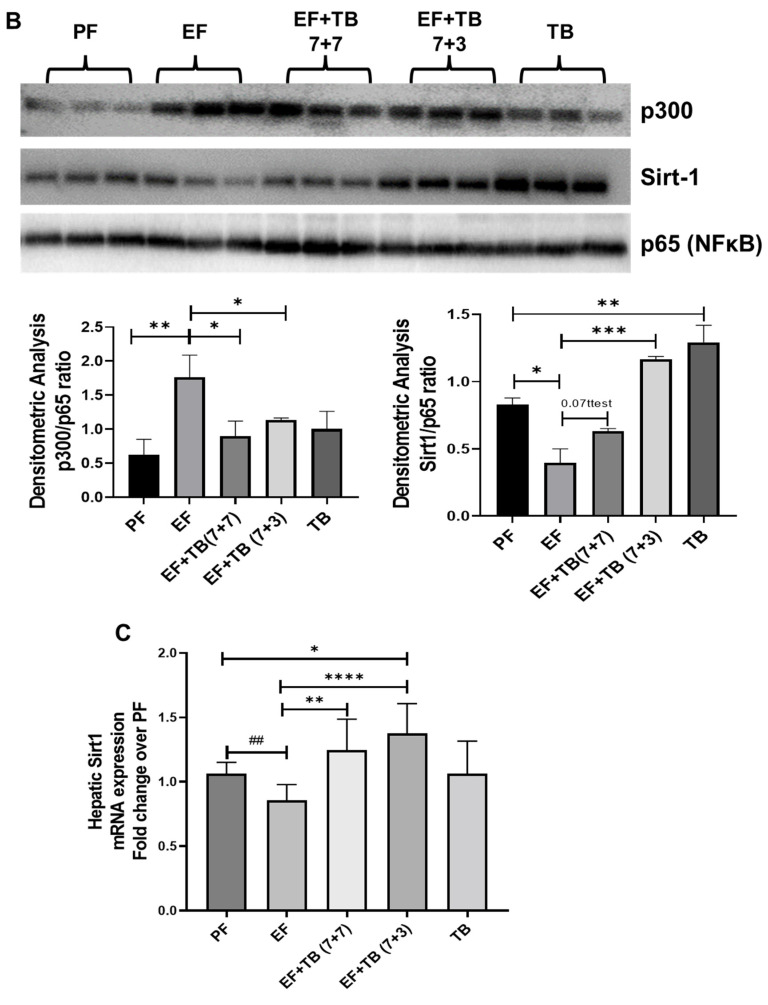
Effect of tributyrin administration on transcriptional activation of NFκB in chronic ethanol-fed mice livers: Mice were fed either a control diet (pair-fed, PF) or ethanol (ethanol-fed; EF) (5% *v*/*v*)-containing diet. Tributyrin (TB; 2 g/kg) was orally administered to ethanol-fed mice as a preventive (EF + TB (7 + 7)) or interventional (EF + TB (7 + 3)) treatment strategy. (**A**) Representative immunoblots for nuclear levels of lysine 310 acetylation (Acetyl-p65-K310) and total p65 subunit of NFκB were shown. The densitometric quantification for Acetyl-p65-K310 after normalizing to total p65 and levels of total p65 after normalizing to Lamine B are shown in the bar graphs. (**B**) Nuclear lysates from mice livers were immunoprecipitated with anti-NFκB-p65 antibodies and immunoblotted with anti-p300, SIRT-1, or NFκB-p65 antibodies. Representative immunoblots are shown. The densitometric quantification was performed, and p300 and SIRT-1 data normalized to NFκB-p65 are shown. (**C**) Hepatic mRNA analysis for SIRT1 was performed via real-time PCR and shown as fold change over PF. Data are presented as mean ± SEM, and a one-way-ANOVA analysis was used; significance is shown as * *p* < 0.05, ** *p* < 0.01, *** *p* < 0.001 and **** *p* < 0.0001. Analysis via Student’s *t*-test is shown as ^##^ *p* < 0.01.

Acetylation of lysine 310 (K310) of the p65 subunit of NFκB is required for its transcriptional activation and DNA-binding affinity [33,34]. Since increased NFκB occupancy was observed upon ethanol feeding, the acetylation status of lysine 310 of the nuclear p65 subunit was examined. Hepatic nuclear lysates were prepared, and Western blot analysis was performed to determine the levels of both total p65-NFκB and acetyl-K310-p65 (Figure 6A). There was a significant increase in the nuclear levels of p65 along with acetylation at K310 in the livers of mice fed ethanol. In contrast, the TB-administered groups showed a decrease in nuclear levels of p65 with a significant reduction in K310 acetylation of p65. These data suggest that TB suppressed the nuclear translocation and transcriptional activation of NFκB that occurred in response to ethanol feeding (Figure 6A).

Importantly, it has been reported that the nuclear acetylation status of K310 is maintained by the interaction of NFκB with a class III histone deacetylase—SIRT1—and histone acetyltransferase p300 [35,36,37]. Hence, the association of NFκB with SIRT1 and p300 was evaluated in ethanol-fed and tributyrin-administered mice. The p65 protein of NFκB was immunoprecipitated from hepatic nuclear lysates, and Western blot analysis was performed to determine the nuclear levels of Sirt1 and p300 associated with p65 (Figure 6B). We observed that chronic ethanol feeding modulated the SIRT1-NFκB-p300 axis in the liver. Specifically, the association of the nuclear RelA/p65 subunit of NFκB with a class III histone deacetylase, SIRT1, was decreased with a concomitant increase in a histone acetyltransferase, p300, interaction in livers of mice chronically fed ethanol compared to controls. On the other hand, TB treatment maintained the SIRT-1 association and reduced the p300 association with NFκB (Figure 6B). We also observed that, as previously shown, ethanol exposure decreased SIRT1 mRNA expression compared to the control. Interestingly, tributyrin, which is known as an HDAC inhibitor, increased hepatic mRNA expression of atypical HDAC-SIRT1 in ethanol-fed mice (Figure 6C).

### 3.7. TB Decreases the Binding of RNA Pol II in Ethanol-Fed Livers

**Figure 7:**
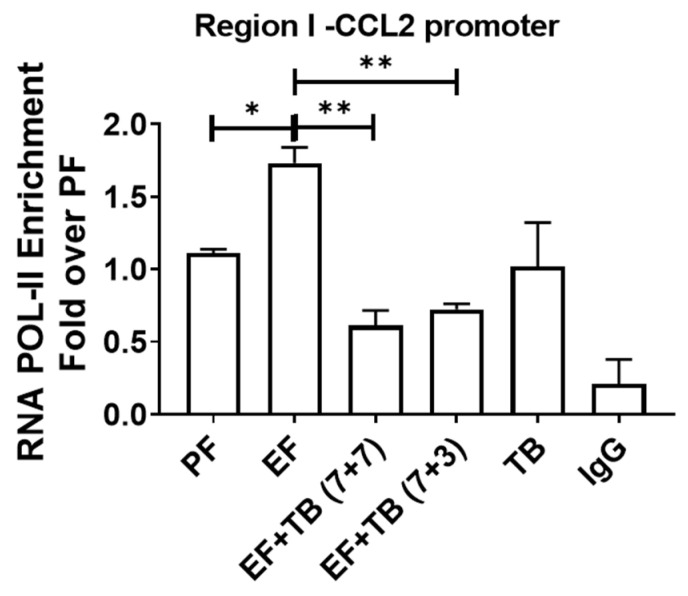
Effect of tributyrin administration on chronic ethanol-induced recruitment of RNA POL II at the CCL2 promoter in the liver: Mice were fed either a control diet (pair-fed, PF) or ethanol (ethanol-fed; EF) (5% *v/v*)-containing diet. Tributyrin (TB; 2 g/kg) was orally administered to ethanol-fed mice as a preventive (EF + TB (7 + 7)) or interventional (EF + TB (7 + 3)) treatment strategy. ChIP-qPCR quantification from immunoprecipitated chromatin using an anti-RNA POL II antibody was performed. Differences are expressed as fold-over PF after normalizing for input DNA. Statistical analysis: mean ± SEM. * *p* < 0.05, and ** *p* < 0.01 compared with PF or EF via ANOVA with Bonferroni’s test (n = 5–8).

In correlation with promoter histone hyper-acetylation and increased NFκB binding, ethanol enhanced the transcription initiation process by recruiting RNA Pol II at the TSS region I of the CCL2 promoter (Figure 7). Importantly, TB prevented these ethanol-induced permissive epigenetic effects and led to a decrease in RNA Pol II recruitment and CCL2 gene transcription (Figure 7).

Taken together, our study indicates that chronic ethanol-induced hyperacetylation of histone H3(K9) and the transcription factor NFκB (K310) drive hepatic CCL2 expression and, in turn, contribute to liver inflammation. Importantly, transcriptional repression of CCL2 via tributyrin administration, under both preventive and interventional treatment strategies, was associated with hypoacetylation and diminished binding of chromatin-associated proteins, including p300, NFκB, and RNA polymerase II.

## 4. Discussion

Alcohol-associated liver disease (ALD) is a major cause of liver-related morbidity and mortality [1,38,39]. Despite being actively investigated, there is no FDA-approved therapy for any stage of ALD. The development of ALD is a complex process that is influenced by a variety of genetic and environmental factors. Research over the past decade has determined that combined effects of alcohol metabolism and compromised nutritional status are associated with disease pathogenesis involving alterations in gut microbiota, key metabolites such as SCFAs, barrier dysfunction, and liver inflammation and injury [18]. In the context of liver inflammation, both clinical and animal studies of ALD have provided evidence that hepatic expression of CCL2 was correlated with the recruitment of macrophages and neutrophils and the severity of liver inflammation and injury [5,7]; however, the mechanisms underlying their upregulation are not completely elucidated. The Ccl2 gene is transcribed at low levels under noninflammatory conditions, and exposure to various pro-inflammatory stimuli/conditions leads to the rapid induction of Ccl2 gene expression [28]. Since the differential Ccl2 gene expression is controlled via an epigenetic mechanism, the present study examined the key aspects of epigenetic regulation of ethanol-inducible CCL2 expression employing the chronic ethanol-feeding mouse model of ALD. The major finding of the present study demonstrated that alterations in the post-translational acetylation modification of both histone H3 and transcription factor NFκB by p300 and SIRT1 play an important role in regulating hepatic CCL2 expression in response to both ethanol and oral administration of tributyrin.

Under both normal and disease states, the epigenetic machinery is important for regulating gene expression. Post-translational modifications (PTMs) of proteins are considered the main components of epigenetic regulatory machinery for gene expression and are also broadly accepted as a therapeutic target [40]. Various post-translational modifications of histone H3 can occur, and depending on the type of modification, it creates a euchromatic or heterochromatic chromatin structure at the promoter, thereby affecting gene expression [41]. Histone H3 acetylation always leads to an open chromatin structure supporting transcription factor binding and enhanced gene transcription. In our study, we observed that ethanol feeding increased H3K9Ac levels at the CCL2 promoter, leading to transcriptional activation and CCL2 gene upregulation. This result was in line with previously published studies where an increase in histone H3K9 acetylation at CCL2 has been reported in obesity and fatty liver [42]. Moreover, ethanol exposure is known to increase global H3K9 acetylation levels in rat hepatocytes [43]. Another documented permissive modification of histone H3 is phosphorylation at serine 10. It is known that H3S10ph not only affects H3K9 acetylation but also acts in synergy with it and increases the efficiency of acetylation reactions [44,45]. Studies by Park et al. have shown a reduction in ethanol-induced acetylation after inhibiting the kinases that are known to phosphorylate Histone H3 at serine 10, suggesting a crosstalk between these two adjacent modifications [43]. We also observed a concomitant change in H3K9 acetylation and H3S10 phosphorylation at the CCL2 promoter under ethanol and tributyrin feeding conditions, further supporting their interactive role in regulating gene transcription. Additionally, ethanol-mediated increases in H3K9 acetylation and H3S10 phosphorylation also enhanced the DNA accessibility for transcriptional machinery, as seen by more binding of NFκB and RNA PoL II upregulating CCL2 transcription.

In addition to histones, acetylation of several non-histone proteins, including transcription factors, also plays an important role in regulating their function and, in turn, target gene transcription [46,47]. NFκB is one such transcription factor whose function is affected by the acetylation of lysine residues [33,48]. Importantly, NFκB is a predominant transcription factor regulating CCL2 gene expression [28,29,49]. Among the seven lysine residues (lysine 122, 123, 218, 221, 310, 314, and 315) of NFκB that are known to become acetylated, acetylation of lysine 310 is important because it is required for full activation of NF-kB [34]. Moreover, it has been reported that lysine 310 is acetylated by HAT-p300 [35], and mutation of this lysine residue does not allow functional cooperation of p65 with p300/CBP and thus impairs the transactivation of p65 [34]. In this context, we observed that livers from ethanol-fed mice showed greater interaction between the p300 and p65 subunit of NFκB and an increase in K310 acetylation of p65.

Thus, these data suggest that ethanol exposure enhanced the transcription potential of NFκB, increasing its binding to the CCL2 promoter and consequent CCL2 upregulation. Additionally, it is known that the interaction of NFκB with co-activator proteins, such as p300, allows their recruitment to target gene promoters [50,51]. The p300, being a histone acetyltransferase (HAT), also acetylates the target gene promoters, creating an open chromatin structure that is permissive for gene transcription. Our data also showed a significant increase in p300 binding to the CCL2 promoter, which then caused the hyperacetylation of histone H3K9 in response to ethanol. These observations suggest that under ethanol conditions, p300 mediates both acetylating histone H3 at lysine 9 and NFκB at lysine 310, thus enhancing the transcription of hepatic CCL2 expression. It is known that alcohol metabolism leads to an increase in acetate levels and acetyl COA levels, which is the main substrate for the p300 acetylation function. Although we have not evaluated the levels of acetyl CoA in our study, based on our results, it can be postulated that ethanol metabolism increases the substrate availability of p300 to acetylate both histone H3 and transcription factor NFκB-p65.

In contrast to ethanol, both preventive and interventional treatment strategies of tributyrin administration showed that TB treatment inhibited the ethanol-inducible CCL2 expression. Tributyrin is a triglyceride containing three butyrate moieties that gets rapidly absorbed and hydrolyzed to butyrate [15,52]. We have previously shown that oral administrations of tributyrin prevent alcohol-induced microbial dysbiosis, mainly loss of butyrate-producing bacteria, and can increase hepato-portal circulation of the butyrate, leading to a direct effect on the liver and protect against steatohepatitis in mice [16]. In the context of inflammation, TB has been shown to reduce the expression of pro-inflammatory cytokines, including MCP-1/CCL2, in adipose tissue in high-fat-fed mice [53]. In the present study, we showed that the protective effects of TB are through decreasing ethanol-mediated permissive acetylation modifications of promoter-associated histones and the p65 subunit of NFκB. Although TB is a dietary butyrate prodrug and has been documented to have HDAC inhibitory function, our study demonstrated that TB increases the hepatic expression and functional capacity of atypical HDAC-SIRT1. Sirtuin 1(SIRT1) is a nicotinamide adenine dinucleotide (NAD+, NADH)-dependent class III histone deacetylase, and its role in alcohol-associated fatty liver disease has been well documented [54]. It has been reported that ethanol downregulates SIRT1 expression in the liver, and ethanol-mediated disruption of SIRT1 signaling leads to fat accumulation and inflammatory responses in the liver [55]. In agreement with the earlier studies, our data also demonstrated a significant decrease in SIRT1 expression in ethanol-fed animals; however, TB treatment not only restored the ethanol-induced downregulation of SIRT1 mRNA expression but further increased its expression. Consistent with the documented results, butyrate has been shown to increase SIRT1 expression in the colon and alleviate DSS-induced inflammation in mice [56]. Moreover, relevant to the present work, it has been reported that changes in p65 acetylation status are regulated by the interaction between p65 and SIRT1 and deacetylation by SIRT1 occurs at lysine 310 [37]. Indeed, our data showed that commensurate with its expression, TB also increases the functional capacity of SIRT1 to interact with and deacetylate the p65 subunit of NFκB. Our data showed that the SIRT1 acts as a negative regulator of NFκB function in TB-treated livers as seen by an increased nuclear association between SIRT-1 and p65 with a significant decrease in acetylation at K310 and subsequent decreased recruitment to CCL2 promoter. The present data are consistent with the previous studies documenting that the use of pharmacological agents modulating SIRT1 activity affects the acetylation status of RelA protein at Lys310 and its inflammatory transactivation potential [35]. It was shown in a SIRT1 knockout mouse model that the deletion of SIRT1, hyperacetylated NFκB and increases its activity, thus resulting in the upregulation of pro-inflammatory genes [57].

TB treatment also interferes with p65 binding to p300, which occurs in response to ethanol exposure. This resultant decrease in p300-p65 association likely contributed to further deacetylation of NFκB and reduced recruitment of p300 to CCL2 promoter, decreasing the H3K9 acetylation levels. Thus, oral administration of tributyrin can induce close chromatin structure and transcriptional suppression of CCL2 expression. It has been reported that SIRT1 can physically interact with p300, inactivating its acetyltransferase capacity [58,59]. Hence, it can be easily postulated that TB exerts its protective effect mainly via modulating HAT-p300 and typical HDAC-SIRT-1 interaction with the p65 subunit of NFκB and deacetylating p65 and impairing its recruitment to CCL2 promoter. Since NFκB is a key regulator of several inflammatory processes, downregulation of NFκB transcriptional activation via tributyrin implies that targeting the SIRT1-NFκB-p300 association may provide new therapeutic opportunities not only for the treatment of ALD but also for other inflammatory conditions. However, more research is needed to fully understand the role of these interactions in ALD and determine if targeting them may be an effective strategy for preventing the development and progression of ALD.

## 5. Conclusions

Overall, our data support the idea that not only acetylation of histone H3 at the CCL2 promoter but also acetylation and transactivation of the non-histone protein, NFκB-p65, contribute to alcohol-induced upregulation of CCL2 in the liver.

## Data Availability

Data will be made available upon reasonable request.

## Abbreviations

ALD, alcohol associated liver disease; MCP-1, monocyte chemoattractant protein-1; CCL2, C-C motif chemokine ligand 2; SCFA, short-chain fatty acid; ChIP, chromatin immunoprecipitation; HAT, histone acetyltransferase; HDAC, histone deacetylase; H3K9Ac, histone 3 lysine 9 acetylation; qPCR, quantitative real-time PCR; RNA Pol II, RNA polymerase II; TSS, transcription start site.
